# Supplementary material for: Resolving the full spectrum of human genome variation using Linked-Reads
Source: Genome Res. 2019 Apr;29(4):635–45. doi: 10.1101/gr.234443.118 (PMC6442396; doi:10.1101/gr.234443.118)
Supplement: Supplemental Material [file supp_gr.234443.118_Supplemental_longranger-2.2.zip › longranger-2.2.tar.bz2!/lr2.2-public/lib/stan/cmdstan/stan/doc/api/html/rev_2operators_2operator__greater__than__or__equal_8hpp_source.html]

Stan: src/stan/agrad/rev/operators/operator\_greater\_than\_or\_equal.hpp Source File


|  |  |
| --- | --- |
|  | Stan  2.5.0  probability, sampling & optimization |


- Main Page
- Related Pages
- Namespaces
- Classes
- Files

- File List
- File Members

All Classes Namespaces Files Functions Variables Typedefs Enumerations Enumerator Friends Macros Pages

- src
- stan
- agrad
- rev
- operators

operator\_greater\_than\_or\_equal.hpp

Go to the documentation of this file.

1 #ifndef STAN\_\_AGRAD\_\_REV\_\_OPERATORS\_\_OPERATOR\_GREATER\_THAN\_OR\_EQUAL\_HPP

2 #define STAN\_\_AGRAD\_\_REV\_\_OPERATORS\_\_OPERATOR\_GREATER\_THAN\_OR\_EQUAL\_HPP

3

4 #include <stan/agrad/rev/var.hpp>

5

6 namespace stan {

7  namespace agrad {

8

27  inline bool operator>=(const var& a, const var& b) {

28  return a.val() >= b.val();

29  }

30

40  inline bool operator>=(const var& a, const double b) {

41  return a.val() >= b;

42  }

43

53  inline bool operator>=(const double a, const var& b) {

54  return a >= b.val();

55  }

56

57  }

58 }

59 #endif

stan::agrad::operator>=

bool operator>=(const fvar< T > &x, const fvar< T > &y)

**Definition:** operator\_greater\_than\_or\_equal.hpp:14

stan::agrad::var::val

double val() const

Return the value of this variable.

**Definition:** var.hpp:209

var.hpp

stan::agrad::var

Independent (input) and dependent (output) variables for gradients.

**Definition:** var.hpp:27


---

[ Stan Home Page ]

*© 2011–2014,
Stan Development Team.*
